# Creating a work environment that keeps you happy

**DOI:** 10.1186/s13244-026-02230-7

**Published:** 2026-03-03

**Authors:** Giles Maskell, Sarah Coope

**Affiliations:** 1 Royal Cornwall Hospital, Truro, UK; 2 Former GP. Director of Training, Wild Monday, Litlington, UK

**Keywords:** Stress, Burnout, Radiology

## Abstract

**Abstract:**

Reports of work-related stress among healthcare workers in all disciplines have increased sharply in recent years, and radiology is no exception. Rising demand, relentless pressures and reduced time for meaningful human contact are all contributing. Against this backdrop, it is more important than ever that we recognise the characteristics of a happy radiology department and then consider strategies which we can deploy as individuals to keep us happy and healthy at work.

**Critical relevance statement:**

The article discusses the importance of a healthy radiology department culture and describes the strategies which an individual radiologist can employ to maximise their own wellbeing in the face of relentless, increasing workload pressure.

**Key Points:**

- The frequency of burnout is increasing among radiologists.
- A happy work environment is essential for providing optimal patient care.
- There are strategies which an individual radiologist can employ to maintain their own well-being in the face of relentless increasing pressure.

**Graphical Abstract:**

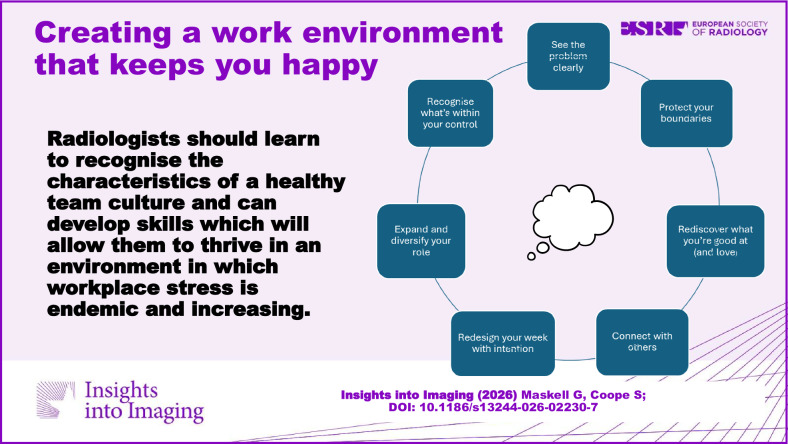

## Introduction

Radiology offers a fascinating, intellectually rich career, yet many radiologists are struggling with burnout, stress and a growing sense of disconnection. Studies conducted across multiple countries and healthcare systems have found a reported prevalence of burnout amongst radiologists ranging from 34% to 85% [1–3].

Faced with rising demand, relentless workload pressure, and shrinking time for meaningful human contact, even the most committed and caring professionals can find themselves stuck in survival mode. But what if there’s a way not just to survive, but to thrive?

This article explores how radiologists can take control of their working lives. We’ll consider first why it matters to have a happy working environment. We’ll then look at the characteristics of a happy and successful radiology department or team, and finally suggest an approach which an individual radiologist can use to support their own wellbeing. We’ll look at how to identify the issues affecting your wellbeing and sense of purpose at work, understand what’s actually within your power to change, and take practical, sustainable steps to create a happier and more fulfilling work environment.

It is not the intention of this article to provide an academic review of the phenomenon of burnout, but a brief explanation of this concept will set the scene for what follows. The term “Burnout” was first used in this context by Maslach in 1976 [4], and the concept was subsequently elaborated with the development of the Maslach Burnout Inventory in 1981 [5]. In 2019, the World Health Organisation recognised burnout as an occupational phenomenon which could have a negative impact on workers in the workplace [6] and defined it as: a syndrome conceptualised as resulting from chronic workplace stress that has not been successfully managed.

It is characterised by three dimensions:

- Feelings of energy depletion or exhaustion.
- Increased mental distance from one’s job, or feelings of negativism or cynicism related to one’s job.
- Reduced professional efficacy.

In more recent writing, Maslach has developed the idea that burnout results from a breakdown or “mismatch” in the relationship between the worker and their job. Factors which might contribute to this mismatch include work overload, lack of control, insufficient rewards, breakdown of community, absence of fairness or value conflicts [7].

## Why does happiness matter at work?

Being happy is a laudable aim in itself. Nobody goes to work wanting to be miserable. But that’s not all. Happy staff are more productive and work for longer. We are all aware of the global workforce crisis in radiology. Recent figures from the Royal College of Radiologists in the UK [8] show that since 2020, the median age of radiologists leaving the National Health Service (NHS) workforce has dropped from 55 to 50. Although these individuals may not be leaving the workforce entirely, this trend demonstrates at least a significant dissatisfaction with their current employment. Their departure represents a worrying loss of knowledge and experience from the hospital radiology service and, of course, puts further pressure on those colleagues who remain, and this at a time when demand for our services is rising sharply. We should be exploring every possible way to support colleagues to continue to contribute.

And most importantly, there is a growing body of evidence that patient outcomes are linked to staff morale. Patients receive better care from happy and motivated staff [9, 10]. Conversely, studies conducted across a range of healthcare disciplines have shown an association between staff burnout and reduced engagement with safety protocols, resulting in increased rates of medical error [11–13]. We are not aware of direct evidence linking radiologist burnout to increased rates of radiological error, but such a link seems highly likely.

## What are the characteristics of a happy radiology department?

The features which distinguish engaged and high-performing teams from others have been studied extensively in industry [14], and many of the findings translate well to healthcare in general and to radiology in particular.

### Regular contact with other team members

More and more radiologists are choosing to work at least some of the time remotely from home or even exclusively in a teleradiology environment. There are sound reasons for this, including a desire for insulation from the interruptions which are inherent in hospital-based activity and also the greater autonomy and control which this mode of working can provide, something we will discuss below. Having said that, the sense of belonging to a team with shared aspirations and objectives has been shown to be a powerful motivating factor, and this is generally enhanced by consistent touchpoints, whether in formal meetings or just a shared coffee break. It is noticeable that a number of teleradiology companies strive to recreate as far as possible for their dispersed reporting workforce the atmosphere of a traditional radiology department with opportunities for shared learning and social events.

### Collective ownership of the service

One of the best outward signs of a well-functioning radiology department is a sense of collective ownership of the whole service. In other words, individual radiologists take pride in and, where possible, support the delivery of aspects of the service which are not their primary concern. This becomes more difficult with increasing department size and specialisation, but in a well-functioning department, most, if not all, radiologists will make some contribution to the emergency workload and will be prepared to make adjustments to their working patterns at short notice, for example, when cover is required for sickness absence.

### Feedback

Across a wide range of activities, it has been found that performance improves with feedback [15], and radiology is no different. If we never get to find out whether our interpretations are right or wrong, we miss the opportunity to improve. It often feels as though most of the feedback we receive is negative—some colleagues are very good at telling us when we get things wrong—and this can provide valuable learning, but is not particularly good for our morale. Mechanisms such as the Royal College of Radiologists’ REALM [16] enable shared learning from errors in a non-threatening and supportive environment and require engagement from all radiologists. The provision of positive feedback is one of the most powerful—and least utilised—tools in healthcare management. The systematic provision of positive feedback is sadly all too rare.

### Interactions between team members

Central to the culture of any department is the way in which individual radiologists interact with each other and with other members of staff. Mutual respect for the knowledge and skills of all team members and for the contributions which all make to the collective enterprise is essential if a happy department atmosphere is to be established and maintained. Patrick Lencioni has explored in the world of business the different characteristics which each of us brings to the working environment and also why some teams are not cohesive [17, 18].

### Leadership

A healthy department or team culture does not arise by accident but invariably requires good leadership. It is outside the scope of this discussion to explore the nature of good leadership, but one of its key characteristics is to exhibit and to demand respectful and mutually supportive behaviours between team members.

## What can an individual do?

In the remainder of this article, we’ll suggest an approach which any radiologist can take to maintain their own well-being in the face of pressures which can seem relentless.

### Seeing the problem clearly

It’s hard to fix what you haven’t yet named. Many doctors describe a low-level but persistent dissatisfaction at work - something they can’t quite pinpoint. That’s why the first step towards creating a happier work life is simply this: stop and take stock.

## The hidden impact of current workloads

Exhaustion has become so common in healthcare that it’s often accepted as the norm. Radiologists are not exempt from this general trend and face particular pressures, exacerbated by never-ending reporting worklists, sometimes unreliable IT systems, and reduced peer interaction. The physical isolation of radiology reporting, coupled with the need for sustained and intense mental concentration, makes emotional burnout even more likely.

## Busyness as a badge of honour

In modern medicine, being overwhelmed is sometimes worn as a badge of honour. The belief that relentless productivity equates to success is deeply ingrained. But this mindset can trap clinicians in cycles of reactive working - rarely pausing to ask whether this pace is sustainable, or even necessary.

## A simple reflection

Try asking yourself:

- How does my current way of working affect me as a person?
- What do I feel at the start—and end—of a typical day?
- Have I stopped enjoying the parts of the job I used to love?

These questions can unearth important clues about where changes are needed.

### Recognising what’s within your control

When work feels overwhelming, it’s tempting to conclude that nothing can change. But while not everything is within your power, far more is possible than many assume.

## Focus on the controllables

Many workplace stressors—like staffing levels, hospital politics or national policies—are beyond your direct control. But the way you respond to those pressures, how you set boundaries, manage your energy, and prioritise your time? That’s yours to shape.

A helpful mindset shift is to separate out:

- What I can change
- What I can influence
- What I need to accept

This approach moves you from passive frustration to empowered action.

## Reframing success

For many of us, professional identity is tightly tied to achievement. The idea that “I am what I do” or “I am only valuable if I’m productive” drives constant striving—at the expense of wellbeing. Letting go of those internal narratives opens space for a more human version of success: one rooted in connection, meaning, and personal alignment.

### Taking action to build a happier work life

Once you’ve identified the issues and your sphere of control, the next step is to take deliberate, focused action. This doesn’t require dramatic career changes. Small shifts, repeated consistently, can transform your experience of work.

#### Redesign your week with intention

Most clinicians don’t actively design their working week—it just happens. But proactively shaping how your time is spent can be game-changing.

- Built-in recovery time: regular short breaks are essential to maintain concentration, as well as to allow for a mental reset.
- Protect focus time: interruptions are toxic to high-quality radiology reporting. Find time and space to allow you to report without interruption, especially for complex cases.
- End your day consciously: set a ritual (like writing tomorrow’s top three priorities) to mark the shift from work to home.

It’s not about being perfect—it’s about being purposeful. Designing your week with purpose can help you regain a sense of control.

#### Expand and diversify your role

Although it may seem counterintuitive, taking on extra tasks can actually increase job satisfaction as long as they align with your interests and values. If your day is filled with only reporting and admin, it’s easy to feel disengaged—even if you’re excellent at it. Many radiologists find renewed energy through small expansions of their role, such as teaching and mentoring, or exploring areas of interest, such as sustainability, equality, or Artificial intelligence. There is no shortage of possibilities.

Adding even 5–10% of work that energises you boosts motivation and reduces burnout.

#### Connect with others

One of the biggest predictors of burnout in medicine is poor quality of connection with others. Yet the practice of radiology is often isolating. Reintroducing regular, intentional interaction with colleagues can make a profound difference.

Options include:

- Forming a peer support group (even informally over coffee).
- Starting reflective practice sessions with colleagues.
- Prioritise corridor chats or brief check-ins with your team.

It doesn’t have to be formal or time-consuming—it just needs to be real. If you are finding it tough at work, you can be sure that you’re not the only one. Paying attention to the well-being of colleagues can pay dividends for you too.

#### Protect your boundaries

Saying no is hard—especially in healthcare. But without boundaries, your time and energy will be drained by things that matter less to you.

Here are some simple, powerful boundary-setting phrases:

- “I’d love to help, but I need to protect my reporting capacity this week.”
- “This needs some time and careful consideration—please put the question in an email, and I’ll get back to you.”
- “I’m not the best person for this—have you tried asking [X]?”

Setting boundaries isn’t being difficult—it’s being sustainable.

#### Rediscover what you’re good at (and love)

When was the last time you reflected on your strengths?

Take a few minutes to list:

- Tasks that energise you.
- Moments when you feel “in flow”.
- Compliments or feedback you’ve received that felt true.

These are often overlooked, but they hold vital clues about where your future satisfaction lies.

Many radiologists keep a record of their errors for future reflection, but equally, we should keep a record of our notable successes—great spots, sound recommendations, the times we used our skills to bring about a better outcome for patients.

## Final thoughts

Creating a happy work environment as a radiologist certainly doesn’t have to be about escaping your job. It’s about crafting it with intention, based on your values, needs, and strengths.

It’s often about choosing courage over autopilot; courage to take back control of the things within your power. Choosing human connection over isolation. And fulfilment over frantic achievement.

We leave you with a question from the poet Mary Oliver:

“…what is it you plan to do with your one wild and precious life?”

In medicine, we often talk about surviving. But you deserve to thrive. And you can start small and take one step at a time to make a difference for you.

Further resources and practical tools to support wellbeing and career development in healthcare can be found at:

www.wildmonday.co.uk

www.youarenotafrog.com

## Data Availability

Not applicable
